# Efficacy and safety of an antithrombotic regimen for atrial fibrillation patients with acute coronary syndrome or those undergoing percutaneous coronary intervention: a meta-analysis

**DOI:** 10.18632/aging.103359

**Published:** 2020-07-01

**Authors:** Wenqin Guo, Xiehui Chen, Yunling Hao, Qiang Liu, Changnong Peng, Lingyue Zhao, Zongming Feng, Xiaoqing Wang, Huanjun Ruan, Lang Li

**Affiliations:** 1 Department of Cardiology, Fuwai Hospital Chinese Academy of Medical Sciences, Shenzhen, China; 2 Department of Ambulatory Surgery, Shenzhen Nanshan District People’s Hospital, Shenzhen, China; 3 Department of Cardiology, The First Affiliated Hospital of Guangxi Medical University, Nanning, China

**Keywords:** antithrombotic regimen, atrial fibrillation, acute coronary syndrome, percutaneous coronary intervention

## Abstract

This study evaluated the benefit of dual therapy in reducing ischemic events in atrial fibrillation (AF) patients presenting with acute coronary syndrome (ACS) or undergoing percutaneous coronary intervention (PCI). We searched PubMed, Cochrane Library, and ClinicalTrials.gov for randomized controlled trials (RCTs) comparing dual and triple therapies (oral anticoagulation plus aspirin and P2Y12 inhibitor) for AF patients with ACS or those undergoing PCI. The composite primary outcome included all-cause death, myocardial infarction (MI), stent thrombosis (ST), or stroke. Relative risk (RR) and the corresponding 95% confidence interval (CI) was used as the measure of effect size. Four RCTs with 10,969 patients were included. Dual therapy had a higher event rate of primary outcome than triple therapy (RR, 1.15; 95%CI, 1.03–1.28; P<0.0001). Dual therapy was associated with significantly higher MI risk, insignificantly higher ST risk, and significantly lower major bleeding risk than triple therapy (RR1.23, 95%CI 1.01–1.49, P = 0.036; RR 1.43, 95 %CI 0.98–2.09, P = 0.064; and RR0.58, 95%CI 0.45–0.76, P<0.0001, respectively). Dual antithrombotic therapy was associated with higher ischemic risk but lower major bleeding risk than triple therapy. The data suggest that antithrombotic regimens should be based on tradeoffs between ischemia and bleeding risk.

## INTRODUCTION

Approximately 5–8% of patients undergoing percutaneous coronary intervention (PCI) have concomitant atrial fibrillation (AF) [1, 2]. Oral anticoagulation can prevent ischemic strokes in high-risk AF patients [3], while aspirin plus P2Y_12_ inhibitor can reduce major adverse cardiovascular events in patients presenting with acute coronary syndrome (ACS) or those undergoing PCI [4]. Triple therapy with anticoagulation plus aspirin and a P2Y_12_ inhibitor is a standard regimen to minimize ischemic events for high-risk AF patients presenting with ACS or undergoing PCI.

Using dual therapy with an oral anticoagulant plus a P2Y_12_ inhibitor for AF patients presenting with ACS or undergoing PCI was based on the hypothesis that oral anticoagulants could replace aspirin for reducing the incidence of ischemic events. Some studies have evaluated the safety and efficacy of dual therapy with oral anticoagulation plus a P2Y_12_ inhibitor for AF patients presenting with ACS or undergoing PCI. The results suggested that dual therapy was associated with a lower risk of bleeding than was triple therapy [5–7]. However, these studies had limited statistical power to evaluate the efficacy profile of dual therapy.

Therefore, we performed a meta-analysis to compare the safety and efficacy of dual therapy (oral anticoagulation plus a P2Y_12_ inhibitor) and triple therapy (oral anticoagulation plus aspirin and a P2Y_12_ inhibitor) in AF patients with ACS or undergoing PCI.

## RESULTS

**Figure 1:**
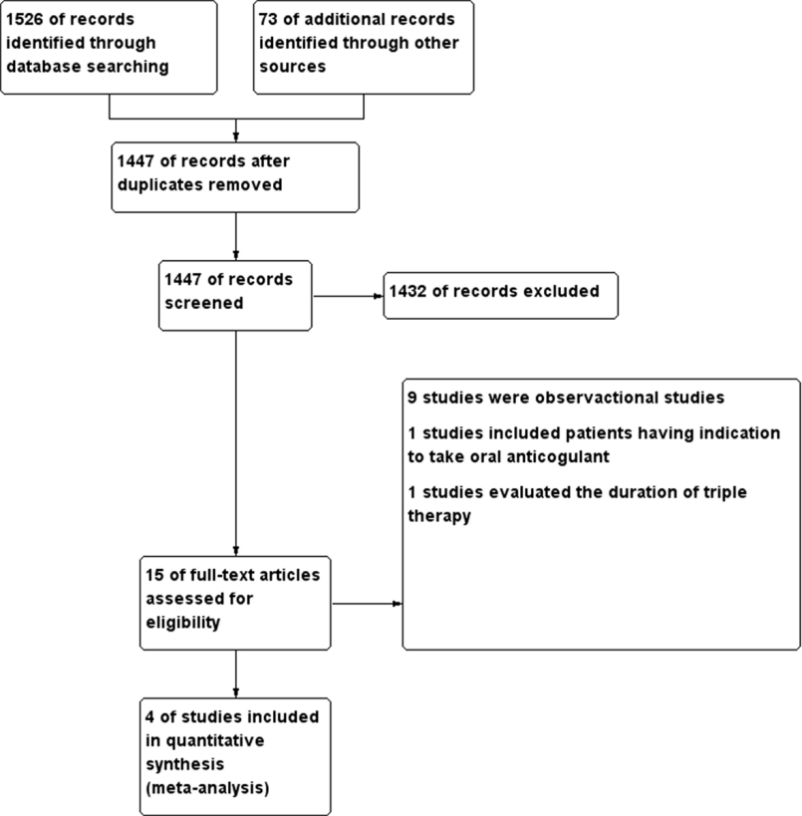
**The literature search and selection.**

The flow chart for study selection is shown in Figure 1. We identified 1,526 studies after searching the databases. We read the references of the included studies and other meta-analysis or reviews on this topic, and another 73 studies were obtained. We excluded 1,432 studies after reading the abstract because they did not meet the inclusion and exclusion criteria. Finally, four RCTs with 10,969 patients were included [5–8]. We excluded the WOEST study because only 70% of the population had AF [9]. The characteristics of the patients at baseline were well-balanced (Table 1). The prevalence of males, diabetes, and ACS were similar among the four trials. The mean age, times in the therapeutic range, target INRs, CHA2DS2-VASc scores, and HAS-BLED scores were also similar. Nearly 50% of patients had ACS. Nearly 90% of the patients received clopidogrel. In the REDUAL study, patients in the dual antiplatelet group were treated with 110 or 150 mg dabigatran etexilate [6]. In the AUGUSTUS study, patients in the dual and triple therapy groups received 5 or 10 mg apixaban [7]. The follow-up time in the AUGUSTUS study was only 6 months [7], while the other studies followed subjects for more than 12 months [5, 6, 8]. An assessment of the risk of bias of the included studies is shown in Supplementary Figure 1. Overall, the included studies had a low risk of bias.

**Table 1:** The characteristic of the included studies.

	**RE-DUAL PCI**	**PIONEER AF-PCI**	**AUGUSTUS**	**ENTRUST-AF PCI**	**P value**
Publish year	2017	2016	2019	2019	NA
Patients	AF patients undergone PCI	AF patients undergone PCI	AF patients with an ACS or undergone PCI	AF patients Undergone PCI	NA
Number of patients	2725	2124	4614	1506	NA
Dual therapy	Dabigatran 110 mg+ P2Y12 inhibitor; Dabigatran 150mg+P2Y12 inhibitor	Rivaroxaban 15 mg +P2Y12 inhibitor	Apixaban(5mg/10mg) /VKA+P2Y12 inhibitor	Edoxaban 60mg+P2Y12 inhibitor	NA
Triple therapy	dose-adjusted VKA+DAPT	Rivaroxaban 2.5 mg +DAPT; dose-adjusted VKA+DAPT	Apixaban (5mg/10mg) /VKA+DAPT	dose-adjusted VKA+DAPT	NA
Follow-up time (months)	14	12	6	12	0.392
Mean Age (years)	70	70	71	69	0.683
Male sex (%)	76	74	71	74	0.572
Creatinine clearance (ml/min)	79.2	78.8	NA	72	NA
P2Y_12_ inhibitor at baseline	Clopidogrel (88%)	Clopidogrel (94.4%)	Clopidogrel (92.6%)	Clopidogrel (92.3%)	NA
	Ticagrelor (12%)	Ticagrelor (4.3%)	Ticagrelor (6.2%)	Ticagrelor (7.0%)	
		Prasugrel (1.3%)	Prasugrel (1.1%)	Prasugrel (0.5%)	
Patients with ACS (%)	50	52	61	52	0.079
Diabetes mellitus (%)	37	30	36	34	0.538
The time in the therapeutic Range (%)	64	65	59	63	0.369
Target INR	2-3	2-3	2-3	2-3	NA
CHA_2_DS_2_-VASc score	3.6	3.8	3.9	4.0	0.850
HAS-BLED score	3.6	3.0	2.9	3.0	0.041

ACS denote acute coronary syndrome, INR international normalized ratio, AF atrial fibrillation, PCI percutaneous coronary intervention, RR risk ratio, VKA vitamin K antagonist, and NA not applicable. Participant in dual therapy group were assigned to receive anticoagulation (including vitamin K antagonist or non–vitamin K antagonist anticoagulants) plus P2Y_12_ inhibitor (including clopidogrel, ticagrelor, or prasugrel), those in triple therapy group were assigned to receive anticoagulation plus aspirin and P2Y_12_ inhibitor.

### Composite endpoint of all-cause death, MI, ST, or stroke

**Figure 2:**
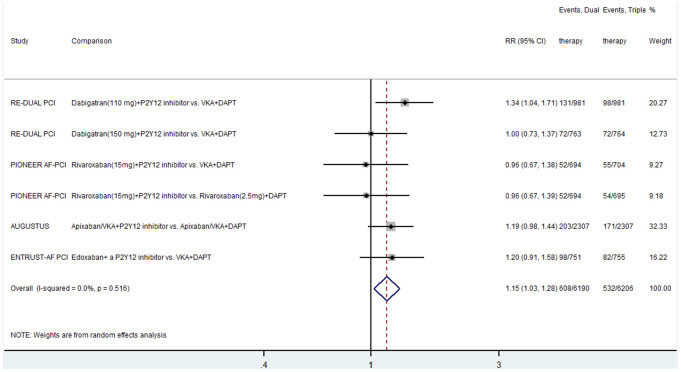
**Meta-analysis of the composite outcomes of all-cause death, myocardial infarction, stent thrombosis and stroke.** Horizontal lines represent the 95% CI of the effect size; solid square indicate the mean effect size in single studies; hollow diamond shapes depict the summary effect size (diamond center) and the relative 95% CI (lateral edges); the black vertical lines represent the reference “1” line.

Four studies compared dual therapy and triple therapy in terms of the composite endpoint of all-cause death, MI, ST, and stroke [5–8]. The results showed that the events rate of the composite outcome of all-cause death, MI, ST, or stroke in the dual therapy group were significantly higher than those in the triple therapy group (RR, 1.15; 95% CI, 1.03–1.28; P < 0.0001; I^2^ = 0) (Figure 2). There was no heterogeneity between the included studies.

### Myocardial infarction

**Figure 3:**
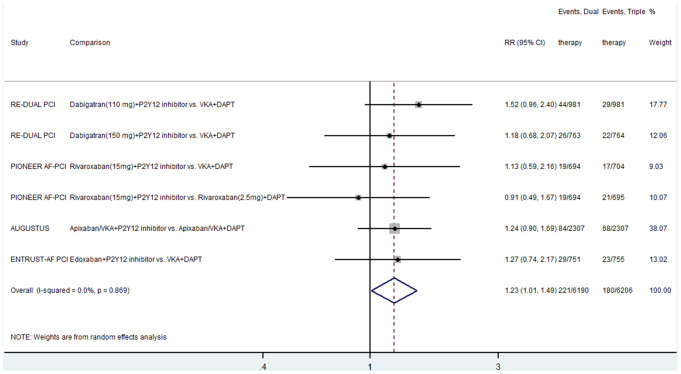
**Results of the meta-analysis of myocardial infarction.** Horizontal lines represent the 95% CI of the effect size; solid square indicate the mean effect size in single studies; hollow diamond shapes depict the summary effect size (diamond center) and the relative 95% CI (lateral edges); the black vertical lines represent the reference “1” line.

Four studies compared the effect of dual and triple therapies on MI [5–8], finding that the event rate of MI was significantly higher in the dual therapy group than in the triple therapy group (RR, 1.23; 95% CI, 1.01–1.49; P = 0.036; I^2^ = 0) (Figure 3). There was no heterogeneity between the included studies.

### Stent thrombosis

**Figure 4:**
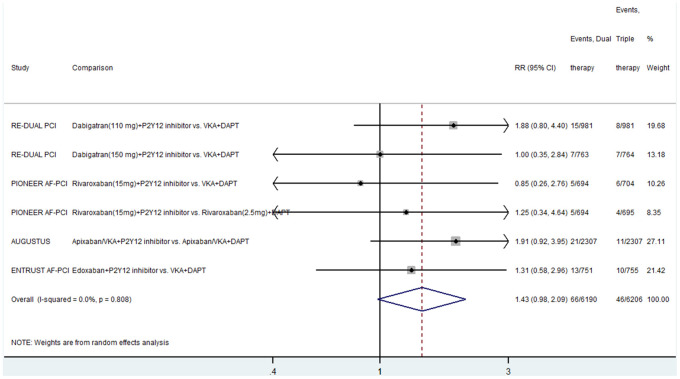
**Results of the meta-analysis of stent thrombosis.** Horizontal lines represent the 95% CI of the effect size; solid square indicate the mean effect size in single studies; hollow diamond shapes depict the summary effect size (diamond center) and the relative 95% CI (lateral edges); the black vertical lines represent the reference “1” line.

Four studies compared the effect of dual therapy and triple therapy on ST [5–8]. The event rate of ST was higher in the dual therapy group than in the triple therapy group, although the difference was not significant (RR, 1.43; 95% CI, 0.98–2.09; P = 0.064; I^2^ = 0) (Figure 4). There was no heterogeneity between the included studies.

### Other clinical outcomes

**Figure 5:**
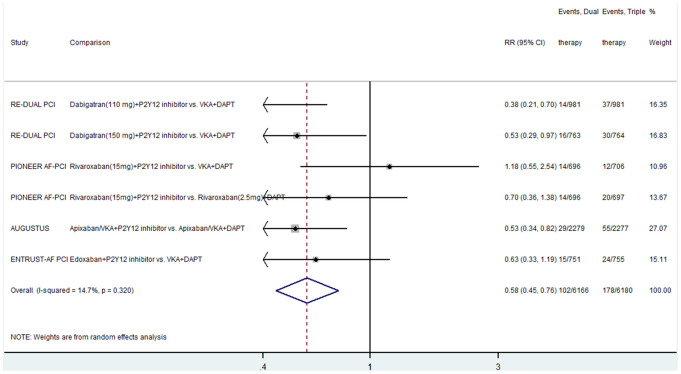
**Results of the meta-analysis of major bleeding.** Horizontal lines represent the 95% CI of the effect size; solid square indicate the mean effect size in single studies; hollow diamond shapes depict the summary effect size (diamond center) and the relative 95% CI (lateral edges); the black vertical lines represent the reference “1” line.

The event rates of all-cause death (RR, 1.06; 95% CI, 0.89–1.27; P = 0.495; I^2^ = 0), cardiovascular death (RR, 1.12; 95% CI, 0.85–1.48; P = 0.420; I^2^ = 0), and stroke (RR, 1.02; 95% CI, 0.73–1.41; P = 0.919; I^2^ = 0) were similar between the two antithrombotic regimens (Supplementary Figures 2–4). Four studies compared the effect of dual and triple therapies on major bleeding [5–8]. Dual therapy was associated with a lower event rate of major bleeding than triple therapy (RR, 0.58; 95% CI, 0.45–0.76; P < 0.0001; I^2^ = 14.7%) (Figure 5). There was low heterogeneity between the included studies.

### Other analysis

The primary efficacy outcome was not affected by publication bias (Supplementary Figure 5).

## DISCUSSION

We found that dual therapy gave a higher event rate of the composite outcome of all-cause death, ST, MI, and stroke than did triple therapy. When each endpoint was analyzed individually, dual therapy was associated with a significantly higher risk of MI and an insignificantly higher risk of ST; however, the risk of major bleeding was significantly lower than with triple therapy. The risks of all-cause death, cardiovascular death, and stroke were identical in the two antithrombotic regimens.

Using dual therapy with an oral anticoagulant plus a P2Y_12_ inhibitor for AF patients presenting with ACS or undergoing PCI was based on the hypothesis that oral anticoagulants could replace aspirin to reduce the incidence of ischemic events. Studies assessing the safety and efficacy of the dual antithrombotic regimen suggested that withdrawing aspirin from triple therapy would reduce the bleeding risk without increasing the incidence of ischemic events [5–8]. However, these studies set the efficacy outcomes as the secondary endpoint; therefore, they had limited statistical power to evaluate ischemic outcomes. A study conducted by Lamberts et al. suggested that the risk of coronary death or MI was similar between vitamin K antagonist (VKA) plus aspirin and VKA monotherapy groups in patients with AF and stable coronary heart disease [10]. A recent AFIRE study showed that rivaroxaban monotherapy was not inferior to aspirin plus rivaroxaban in terms of efficacy and was superior in terms of safety [11]. Nevertheless, in patients with ACS or those undergoing PCI, no studies had been performed to evaluate the benefit of oral anticoagulants in reducing the incidence of ischemic events. The results from GEMINI-ACS-1 showed that the bleeding risk of rivaroxaban combined with a single P2Y_12_ inhibitor was similar to that of dual antiplatelet therapy (DAPT) without increasing the rate of ischemic events in patients with ACS [12]. However, this study also lacked the statistical power to evaluate ischemic outcomes. Whether rivaroxaban can replace aspirin to reduce ischemic events in patients after ACS or PCI remains unclear.

We used meta-analysis to synthesize current evidence and found that dual therapy was associated with a higher risk of ischemic events than was triple therapy in AF patients with ACS or those undergoing PCI. Approximately 50% of patients had ACS in our study; therefore, the early discontinuation of aspirin from the traditional triple therapy decreased the bleeding risk but increased the rate of ischemic events among this population at high-risk for ischemia. The antithrombotic regimen for high-risk AF patients with ASC or those undergoing PCI should be based on the trade-off of between risks of ischemia and those of bleeding. For example, triple therapy could be prescribed for patients in whom the ischemic risk outweighs the bleeding risk.

The 2019 AHA/ACC/HRS guidelines for the management of AF included a class IIb recommendation stating that triple therapy for 4–6 weeks may be considered for high-risk AF patients who had undergone PCI with stenting for ACS [13]. The 2017 ESC on DAPT included a class IIa recommendation stating that triple therapy for longer than 1 month and up to 6 months should be considered in patients whose high ischemic risk outweighed their bleeding risk [14]. Therefore, the timing for withdrawal of aspirin from triple therapy for patients with high ischemic risk remains unclear. The status of aspirin in antithrombotic therapy was weakened by recent studies such as the STOPDAPT-2 [15] and SMART-CHOICE trials [16], in which aspirin was discontinued 1–3 months after stent implantation. The results demonstrated that early discontinuation of aspirin was not inferior to continuous DAPT in reducing ischemic events. Nevertheless, most of the patients in these studies were those at low ischemic risk. There remains limited evidence as to the optimal duration of triple antithrombotic therapy, especially for those at high ischemic risk. Therefore, further studies are warranted to determine the optimal duration of triple antithrombotic therapy.

### Limitations of the study

Our study was not without limitations. First, we did not obtain the first time to event of the primary efficacy outcome because this was not available. Nevertheless, we analyzed each endpoint individually, and the results showed that the dual therapy was associated with a higher event rate of primary efficacy outcomes, primarily driven by the significantly increased MI risk and the insignificantly increased ST risk. Second, previous calculations have estimated that approximately 14,000 patients would be needed to provide adequate power for the assessment of ischemic outcomes in patients with AF who present with ACS or undergo PCI [17, 18]. Our study included 10,969 patients, which is the largest sample size at present. However, the number of patients included in this meta-analysis falls short of those figures. Therefore, more studies are warranted to improve the statistical power for the assessment of ischemic outcomes. Third, we could not perform a time-trend analysis of ischemic events; therefore, we could not determine the optimal duration of triple therapy. Fourth, we could not perform subgroup analysis according to the type of P2Y_12_ inhibitor, the HSBLED score, or the CHA2DS2-VASc score. Therefore, a patient-level meta-analysis is warranted to identify those patients who would benefit from dual therapy. Fifth, the benefit of dual therapy for decreasing ischemic risk may have been underestimated because of the insufficient dose of dabigatran etexilate (110 mg). Therefore, further studies to determine the optimal does of new oral anticoagulants are warranted. Sixth, our meta-analysis showed that dual therapy was associated with a non-significantly higher risk of ST than triple therapy. However, the follow-up time in the included studies was not long enough. For instance, the AUGUSTUS study had a follow-up duration of only 6 months [7]. Therefore, an updated meta-analysis with a long follow-up time is needed to evaluate the benefit of dual therapy in reducing ischemic events.

## CONCLUSIONS

Dual antithrombotic therapy was associated with a higher risk of ischemic events but with a lower risk of major bleeding than triple antithrombotic therapy in patients with high-risk atrial fibrillation and undergoing PCI. These findings suggest that the antithrombotic regimen for high-risk AF patients with ACS or those undergoing PCI should be based on the trade-off of ischemic and bleeding risks.

## MATERIALS AND METHODS

We performed this analysis according to The Quality of Reporting of Meta-analyses (QUOROM) Statement. We conducted a literature search using PubMed, Cochrane Library, and ClinicalTrials.gov to identify relevant articles from the inception date of the databases to September 2019; the references of the included studies and other meta-analysis or reviews on this topic were also searched. The following keywords and MeSH terms were used: “atrial fibrillation,” “percutaneous coronary intervention,” and “acute coronary syndrome.”

### Inclusion and exclusion criteria

The inclusion criteria of the study were as follows: 1) high-risk AF patients presenting with ACS or undergoing PCI; 2) the intervention group was dual therapy with oral anticoagulation plus single P2Y_12_ inhibitor; 3) the control group was triple therapy with oral anticoagulation plus aspirin and single P2Y_12_ inhibitor, and 4) the study must be a randomized controlled trial (RCT).

### Outcomes

The primary efficacy endpoint was the composite outcome of all-cause death, myocardial infarction (MI), stent thrombosis (ST), or stroke. The secondary efficacy endpoints were all-cause death, cardiovascular death, MI, ST, and stroke. All efficacy endpoints were defined according to each trial's criteria. The primary safety outcome was major bleeding, defined according to the Thrombolysis in Myocardial Infarction definitions.

### Data extraction and study quality assessment

We produced a table in which two researchers (W-QG and Z-MF) independently extracted the following information from the studies: publication time, the comparison, reported endpoints, mean follow-up time, and characteristics of the population (e.g., average age, the proportion of males, diabetes, and ACS). If the information extracted by the researchers differed, the discrepancy was resolved by a third researcher. If the same study had more than one report, we extracted data from the most recent report. The intention-to-treat sample was used when available. The Cochrane risk of bias tool was used to assess the quality of the included studies.

### Statistical analysis

We used the DerSimonian-Laird method to calculate the relative risk (RR) and corresponding 95% confidence intervals (CIs) using a random-effects model. The Cochrane Q test and the inconsistency index (I^2^) test were used to assess statistical heterogeneity. I^2^ values < 25% were indicative of low heterogeneity, whereas I^2^ values between 25% and 50% were indicative of moderate heterogeneity and I^2^ values >50% were indicative of high heterogeneity. The REDUAL-PCI study was treated as two data sets because there are two dosage forms of dabigatran etexilate in the dual antiplatelet group (i.e. 110 mg and 150 mg). The PIONEER AF-PCI study was treated as two data sets because patients in the triple therapy group received warfarin or rivaroxaban as anticoagulants. The funnel plot method and Egger's regression asymmetry test were used to determine if publication bias was present. Meta-analysis was performed using STATA software, version 13.0 (StataCorp, College Station, TX, USA).

## Supplementary Material

Supplementary Figures
