# Supplementary material for: Efficacy and safety of an antithrombotic regimen for atrial fibrillation patients with acute coronary syndrome or those undergoing percutaneous coronary intervention: a meta-analysis
Source: Aging (Albany NY). 2020 Jul 1;12(13):12930–42. doi: 10.18632/aging.103359 (PMC7377825; doi:10.18632/aging.103359)
Supplement: Supplementary Figures [file aging-12-103359-s001..pdf]

SUPPLEMENTARY FIGURES

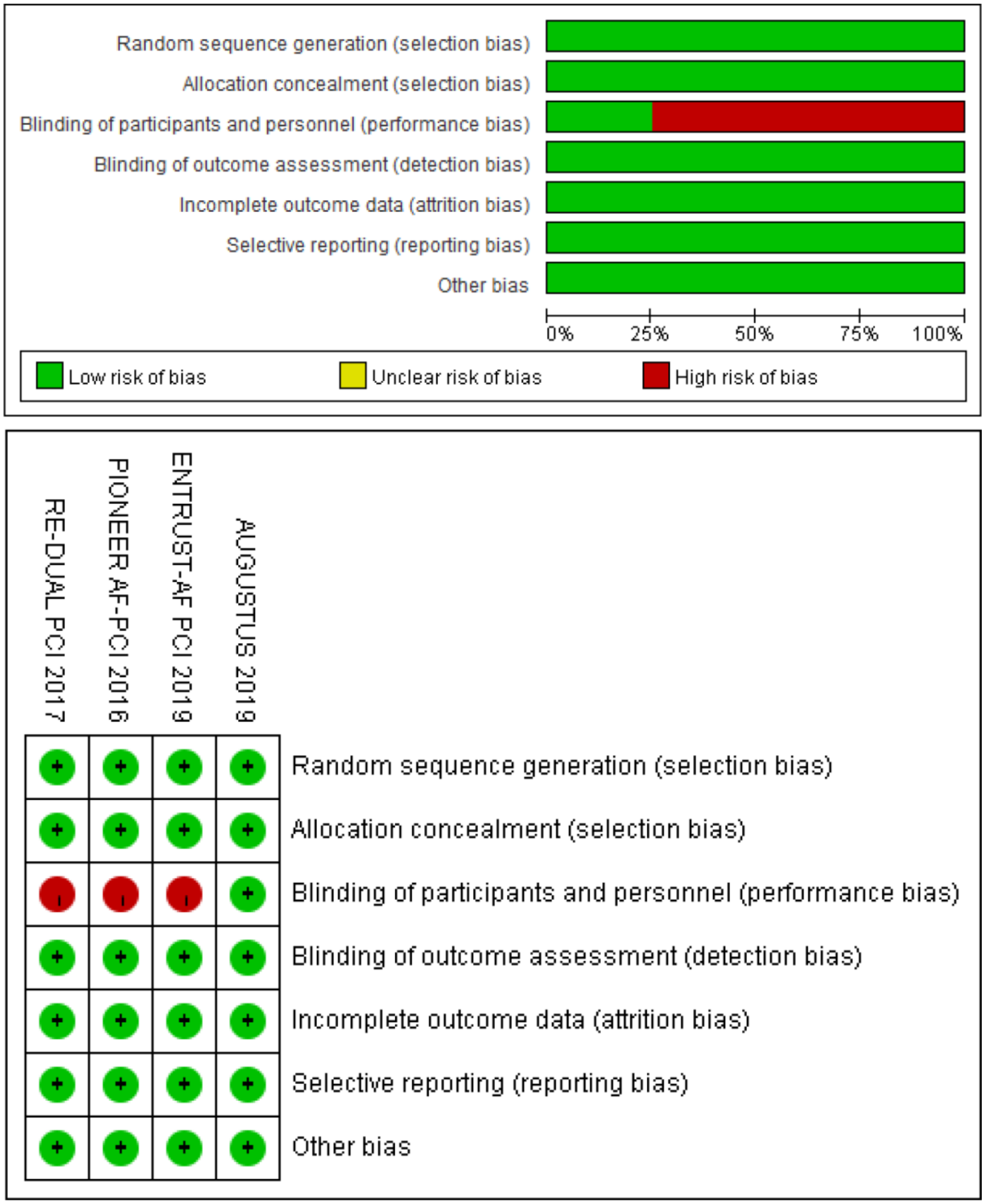

Supplementary Figure 1. Risk-of-Bias assessment of the randomized controlled trials.

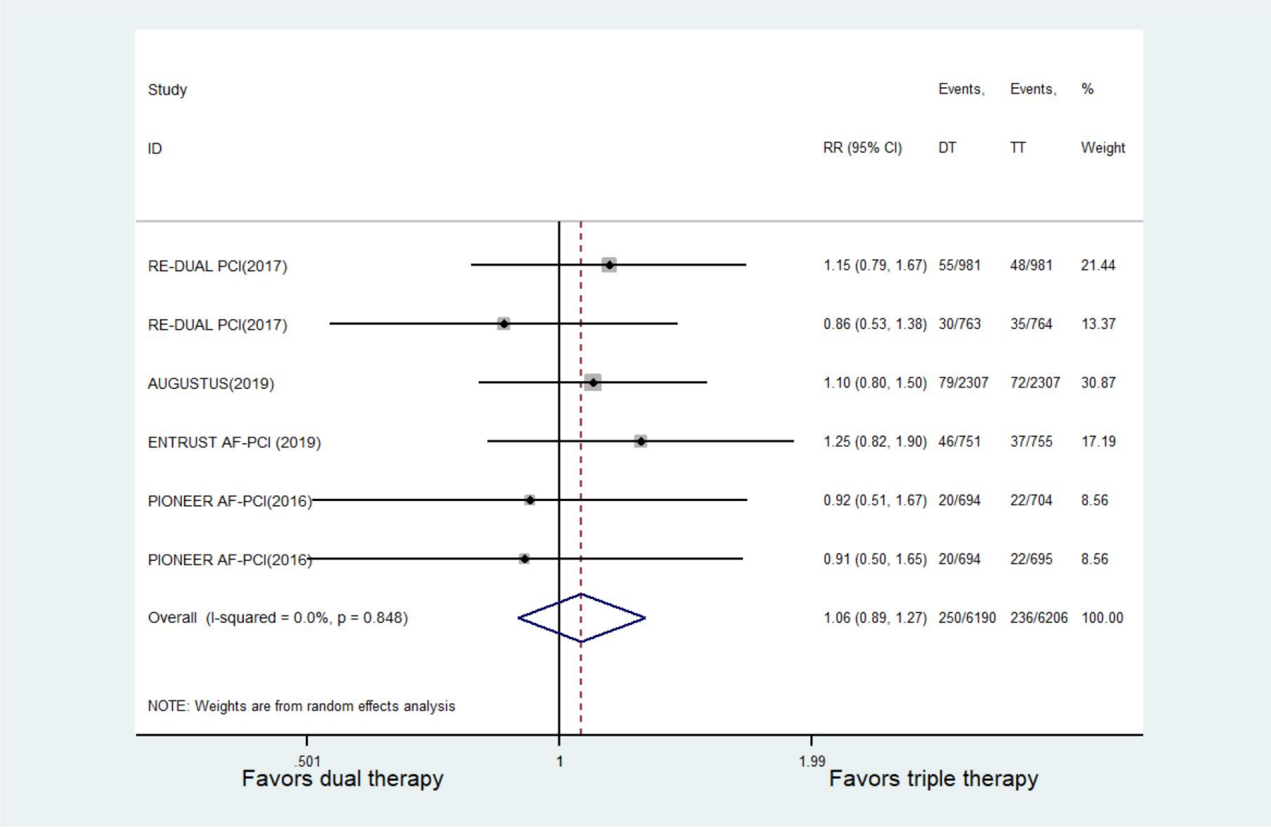

Supplementary Figure 2. Results of the meta-analysis of the all-cause death.

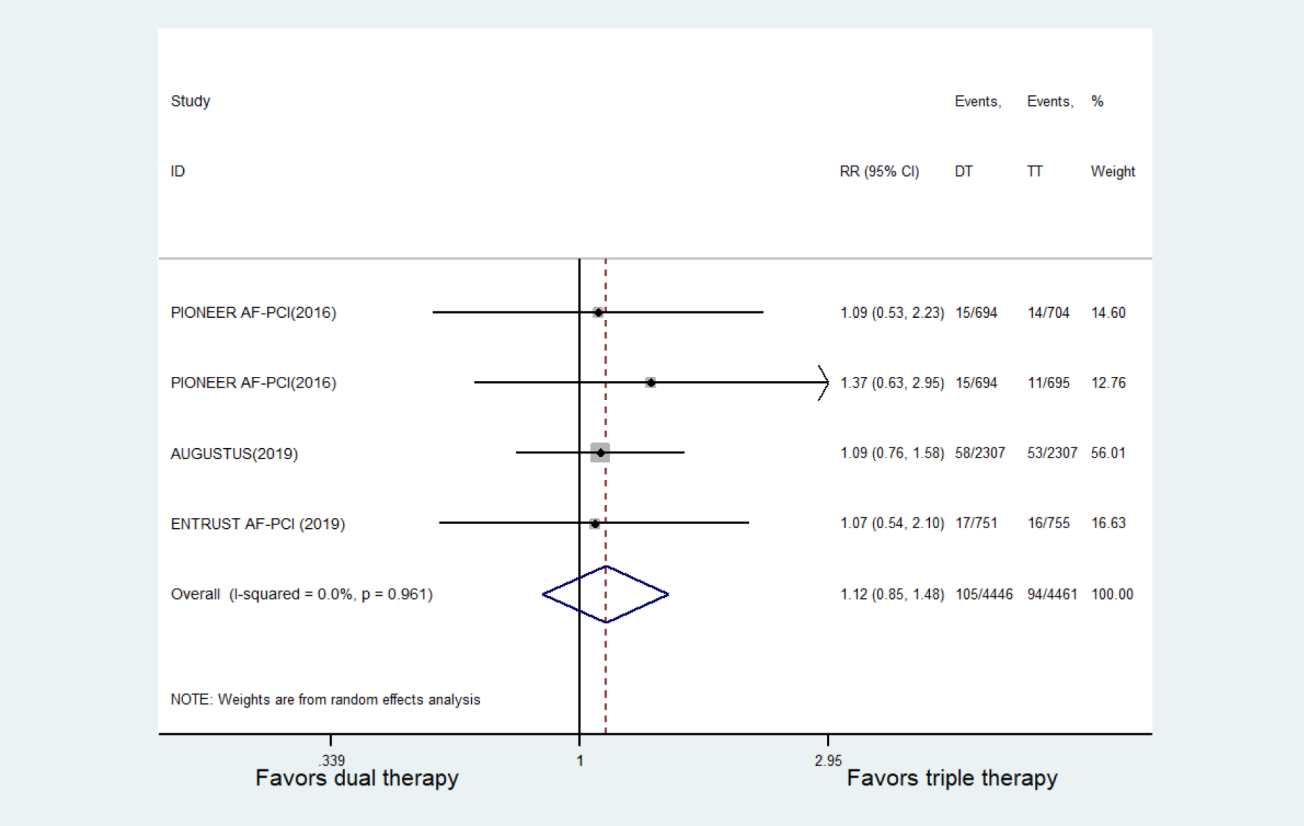

Supplementary Figure 3. Results of the meta-analysis of the cardiovascular death.

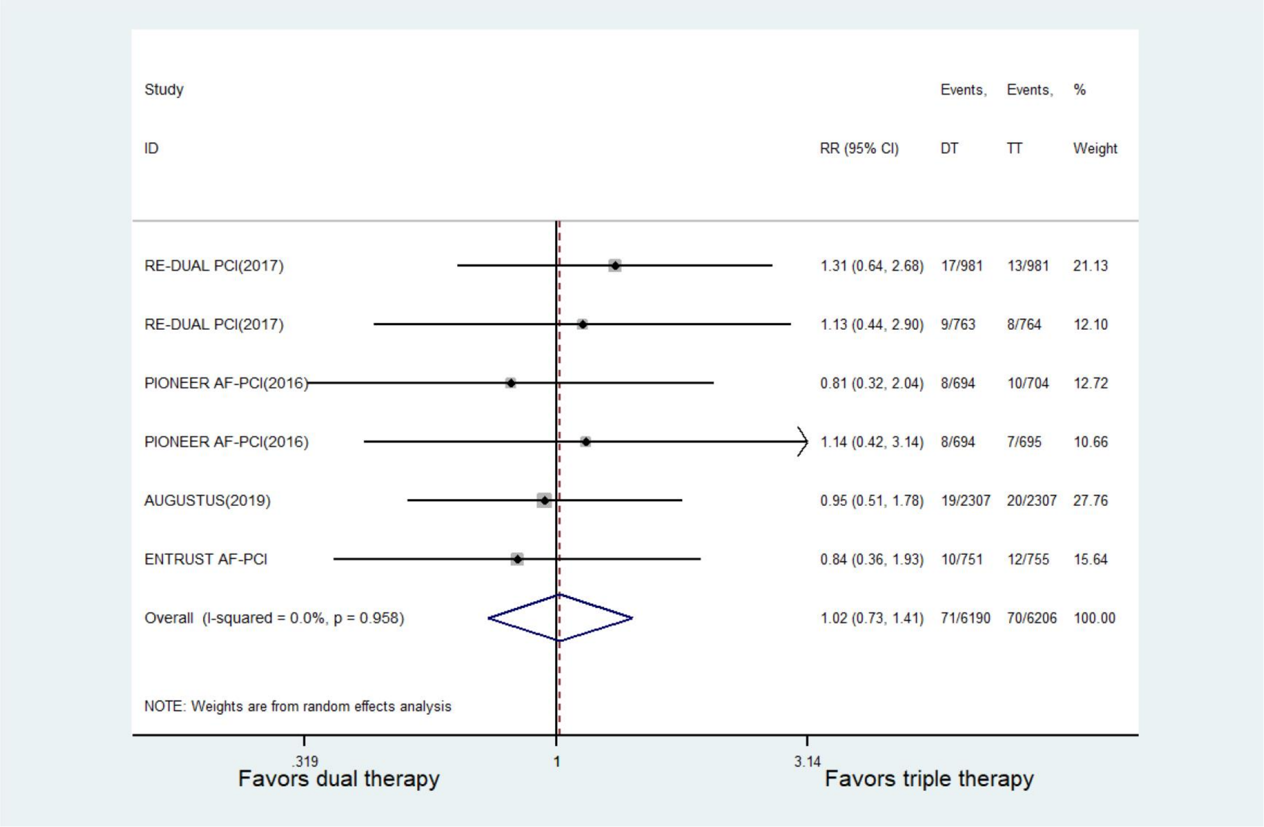

Supplementary Figure 4. Results of the meta-analysis of the stroke.

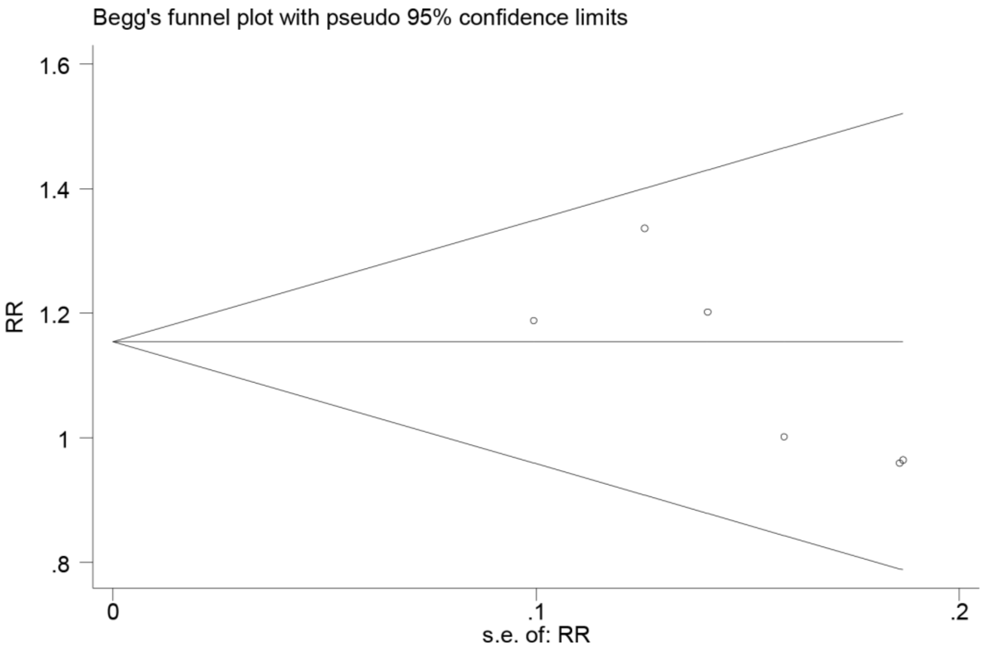

Supplementary Figure 5. The funnel plots.
